# Anti-Diabetic Effect of a Shihunine-Rich Extract of *Dendrobium* *loddigesii* on 3T3-L1 Cells and db/db Mice by Up-Regulating AMPK–GLUT4–PPARα

**DOI:** 10.3390/molecules24142673

**Published:** 2019-07-23

**Authors:** Xue-Wen Li, Meixiang Huang, Kakei Lo, Wei-Li Chen, Ying-Yan He, Yongli Xu, Huizhen Zheng, Haiyan Hu, Jun Wang

**Affiliations:** School of Pharmaceutical Sciences, Sun Yat-sen University, Guangzhou 510006, China

**Keywords:** shihunine-rich extract of *Dendrobium loddigesii*, type 2 diabetes mellitus, db/db mice, 3T3-L1 cells, cleaved caspase-3/AMPK/GLUT4/PPARα, toxic activity

## Abstract

The stems of *Dendrobium loddigesii*, a Chinese herb, are often used to treat diabetes and its polar extract is rich in shihunine, a water-soluble *Orchidaceae* alkaloid, but little is known about the anti-diabetes effects and mechanism of shihunine. This study investigated the anti-diabetic effect of a shihunine-rich extract of *D. loddigesii* (DLS) based on 3T3-L1 cells and db/db mice. The underlying mechanisms were primarily explored using Western blot analysis and immunohistochemical staining. The 3T3-L1 cell experiments showed that DLS can reduce the intracellular accumulation of oil droplets as well as triglycerides (*p* < 0.001) and promote the 2-[*N*-(7-nitrobenz-2-oxa-1,3-diazol-4-yl)amino]-2deoxyglucose (2-NBDG) uptake of 3T3-L1 cells (*p* < 0.001). The animal experiments confirmed that after 8 weeks of DLS treatment, the body weight, fasting blood sugar, and serum lipid levels of mice were significantly lowered, and the oral glucose tolerance test and serum insulin level were significantly improved compared to the no-treatment diabetes mellitus group. Further histomorphology observation led to the conclusion that the quantities of islet cells were significantly increased and the increase in adipose cell size was significantly suppressed. The immunohistochemical test of pancreatic tissue revealed that DLS inhibited the expression of cleaved cysteine aspartic acid-specific protease 3 (cleaved caspase-3). Western blot experiments showed that DLS had agonistic effects on adenosine monophosphate (AMP)-activated protein kinase phosphorylation (p-AMPK) and increased the expression levels of peroxisome proliferator-activated receptor α (PPARα) and glucose transporter 4 (GLUT4) in liver or adipose tissues. These data suggest that the shihunine-rich extract of *D. loddigesii* is an anti-diabetic fraction of *D. loddigesii*. Under our experimental condition, DLS at a dose of 50 mg/kg has good anti-diabetic efficacy.

## 1. Introduction

Type 2 diabetes mellitus (T2DM) is a metabolic disease with a high prevalence, and can seriously damage people’s health [1,2]. The clinical symptoms of T2DM are hyperglycemia, insulin resistance, hyperlipidemia, and a relative deficiency of insulin secretion because of pancreatic β-cell dysfunction [3]. Obesity is the most critical factor in the emergence of metabolic diseases and insulin resistance [4]. Obesity results in insulin resistance and β-cell dysfunction, which are unable to fully compensate for the reduced insulin sensitivity of liver tissue, adipose tissue, and skeletal muscle, triggering T2DM [5,6,7]. Preventing obesity and improving insulin resistance are regarded as one of the important strategies of treating T2DM. 

Adenosine monophosphate-activated protein kinase (AMPK) is an important protein kinase in cell energy metabolism, which is an attractive pharmacological target for T2DM [8,9]. Some AMPK activators such as metformin and thiazolidinediones are used for the treatment of T2DM [9,10,11,12]. Glucose transporter 4 (GLUT4) is an insulin-regulated glucose transporter in the AMPK pathway [13,14]. High expression levels of GLUT4 in adipose tissue can enhance insulin sensitivity, as well as glucose tolerance [15,16]. Peroxisome proliferator-activated receptors (PPARs) are nuclear receptors and play pivotal roles in regulating lipid metabolism [17]. PPARα can decrease plasma triglyceride levels by regulating fatty acid catabolism in liver mitochondria, and control fatty acid utilization [18,19,20]. The function of PPARα might be associated with AMPK [17]; the fatty acid catabolism that occurs by PPARα regulation is thought to be a synergistic effect of AMPK [13,21,22]. 

The stems of *Dendrobium* are used as an anti-diabetic herb in traditional Chinese medicine [23,24,25,26], and there are approximately 76 *Dendrobium* species in China [23]. The main metabolites of *Dendrobium* are alkaloids, polyphenols, and polysaccharides [27]. Shihunine is a water-soluble phthalide-type *Orchidaceae* alkaloid (Figure S1), and one of the five types of structural skeletons of *Dendrobium* alkaloids [27]. Shihunine is the main metabolite of a polar extract of *D. loddigesii* [27,28]. The pharmacological activity of shihunine and the polar extract of *D. loddigesii* have rarely been researched, to the best of our knowledge. In a previous study, we reported the effects of polyphenols of *D. loddigesii* for the treatment of T2DM in db/db mice [29]. To continue our study on anti-diabetic components and mechanisms of *D. loddigesii*, the anti-diabetic effects of a shihunine-rich extract of *D. Loddigesii* were researched in 3T3-L1 cells and db/db mice. 

## 2. Results

The polar extract of *D. loddigesii* is rich in shihunine [27,28] and little is known about its pharmacological activity. To clarify the anti-diabetic effect of *D. loddigesii*, a shihunine-rich extract (DLS) was prepared from *D. loddigesii* and its anti-diabetic effects were researched in 3T3-L1 cells and db/db mice. To probe its related mechanism, the expression levels of cleaved caspase-3, AMPK, GLUT4, and PPARα in the mice tissues were analyzed using immunohistochemistry and Western blot analysis. The toxicity of DLS for C57 mice was preliminarily observed for its safety.

### 2.1. Composition of a Shihunine-Rich Extract of D. loddigesii

A shihunine-rich extract of *D. loddigesii* (DLS) was qualitatively analyzed using ^1^H-NMR, which showed the ^1^H resonance signals of shihunine (Figures S2 and S3) and the signals of carbohydrate in the spectral region from 3.0 to 4.0 ppm (Figure S3). The results showed that the DLS extracts were mainly composed of shihunine and carbohydrate. The purity of shihunine in DLS was evaluated by means of the Quantitative Nuclear Magnetic Resonance (qNMR) method, using salicylic acid as the internal standard. The result showed that the shihunine in DLS displayed a 38% purity by a comparison of the peak areas of *δ*_H_ 8.15 ppm (shihunine) and *δ*_H_ 7.85 ppm (salicylic acid) in the ^1^H-NMR spectrum (Figures S3 and S4). The enrichment factor for shihunine was 165 [28]. 

### 2.2. Effect of a Shihunine-Rich Extract of D. loddigesii on the 3T3-L1 Preadipocyte

#### 2.2.1. Inhibition of Preadipocyte Differentiation and Lipogenesis

**Figure 1:**
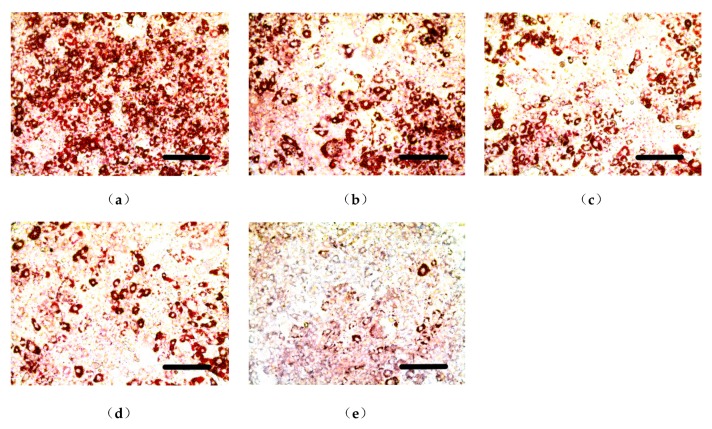
Effect of a shihunine-rich extract of *Dendrobium loddigesii* (DLS) on the differentiation of a 3T3-L1 preadipocyte stained by Oil Red O. Scale bars equal to 200 μm; (**a**) Mod group: fully differentiated 3T3-L1 adipocyte; (**b**–**e**) DLS groups: fully differentiated 3T3-L1 cells treated with DLS at 2.03, 4.06, 8.12, and 16.25 μg/mL, respectively.

**Figure 2:**
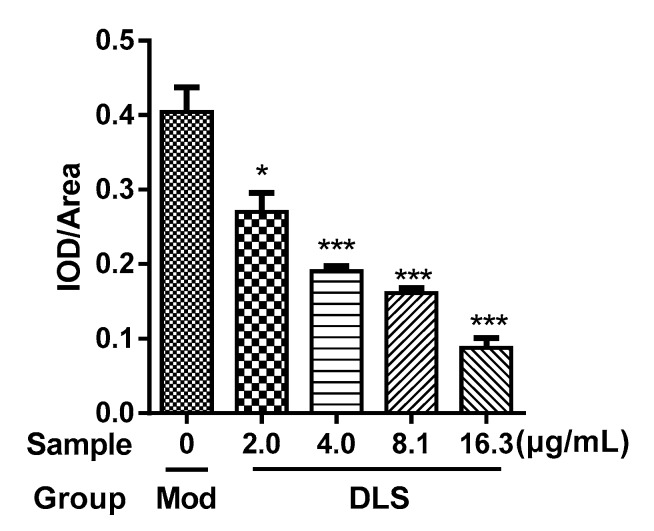
Gray value (integrated optical density (IOD)/Area) of 3T3-L1 cells stained by Oil Red O. Mod group: fully differentiated 3T3-L1 cells; *Dendrobium loddigesii* (DLS) groups: fully differentiated 3T3-L1 cells treated with DLS at 2.03, 4.06, 8.12, and 16.25 μg/mL, respectively; data were mean ± SD; * *p* < 0.05 and *** *p* < 0.001 vs. Mod.

Some inducing agents can induce the differentiation of undifferentiated 3T3-L1 cells. Intracellular fats such as triglycerides (TG) were increased in differentiated 3T3-L1 cells that were stained red by Oil Red O staining [30]. This model was used to study the activity of the decreasing lipid content of a shihunine-rich extract of *D. loddigesii* (DLS). Images of the cells are shown in Figure 1 and the results of gray value analysis are presented in Figure 2.

Figure 1a presents the fully differentiated 3T3-L1 cells, in which a 95% cell volume was occupied by oil droplets. Figure 1b–e show that the accumulation of oil droplets was reduced significantly after being treated with DLS at different concentrations. Figure 2 demonstrates that DLS inhibited oil droplet accumulation in a dose-dependent manner. Compared with fully differentiated 3T3-L1 cells (Figure 1a), the oil droplet contents of DLS groups were decreased by 25% to 80%, with a significance of *p* < 0.05 or *p* < 0.001.

**Figure 3:**
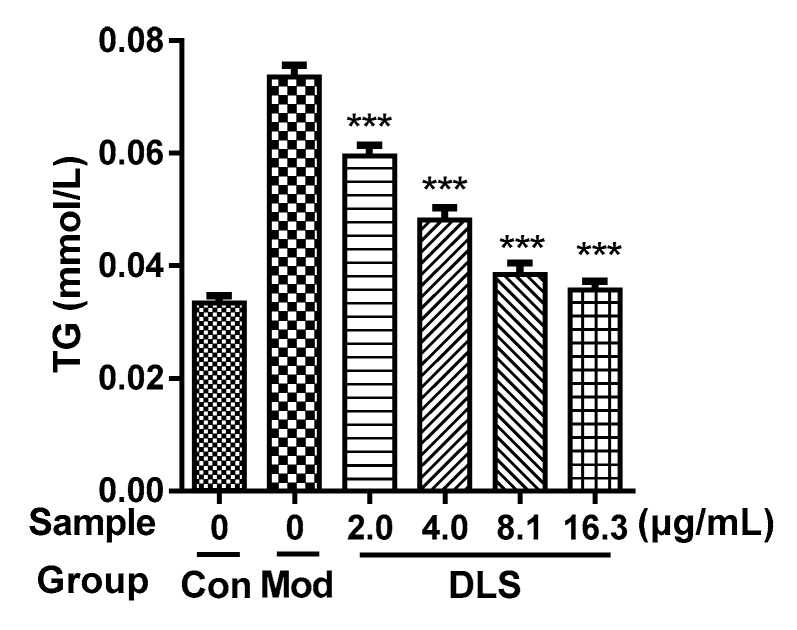
Effect of a shihunine-rich extract of *Dendrobium loddigesii* (DLS) on the triglyceride content of 3T3-L1 cells. Con: undifferentiated 3T3-L1 cells; Mod: fully differentiated 3T3-L1 cells; DLS groups: fully differentiated 3T3-L1 cells treated with DLS at 2.03, 4.06, 8.12, and 16.25 μg/mL, respectively; data were mean ± SD; *** *p* < 0.001 vs. Mod.

The effect of DLS on the inhibition of lipogenesis was studied further. The contents of intracellular TG were determined using enzyme-linked immunosorbent assays (ELISAs) and the result is shown in Figure 3.

As Figure 3 shows, the TG contents of DLS groups were obviously lower than those of the Mod group (*p* < 0.001), which were reduced in a dose-dependent manner. Compared with the Mod group, the TG content of the 16.25 μg/mL group was reduced by 50% (*p* < 0.001).

#### 2.2.2. Improvement of Glucose Uptake

**Figure 4:**
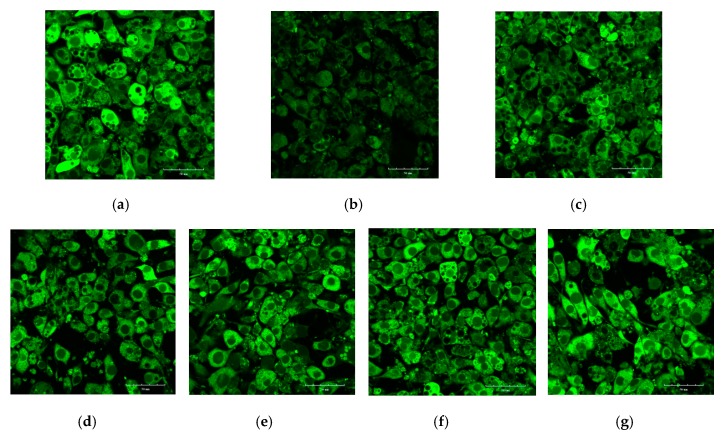
Effect of a shihunine-rich extract of *Dendrobium loddigesii* (DLS) on the 2-[*N*-(7-nitrobenz-2-oxa-1,3-diazol-4-yl)amino]-2deoxyglucose (2-NBDG) uptake of insulin-resistant (IR)-like 3T3-L1 cells by laser confocal scanning microscopy. Scale bars equal to 50 μm; (**a**) Con group: fully differentiated 3T3-L1 cells; (**b**) Mod group: IR-like 3T3-L1 cells; (**c**) positive drug group: IR-like 3T3-L1 cells treated with 3.92 μg/mL pioglitazon; (**d**–**g**) DLS groups: IR-like 3T3-L1 cells treated with DLS at 2.03, 4.06, 8.12, and 16.25 μg/mL, respectively.

**Figure 5:**
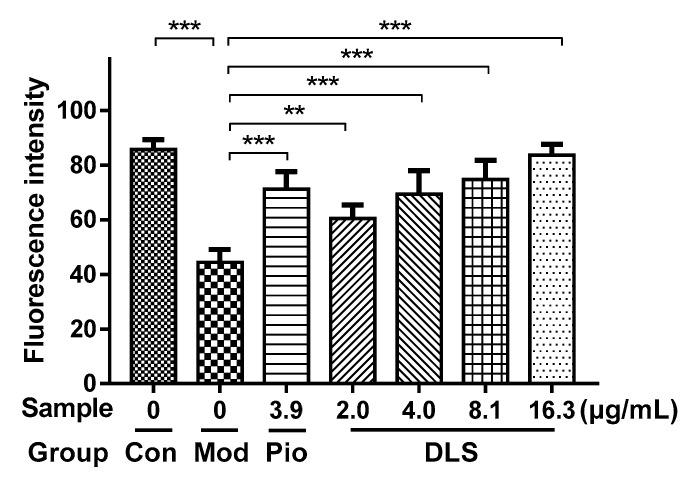
Fluorescence intensity of 2-NBDG in insulin-resistant (IR)-like 3T3-L1 cells; Con group: fully differentiated 3T3-L1 cells; Mod group: IR-like 3T3-L1 cells; Pio group: IR-like 3T3-L1 cells treated with 3.92 μg/mL pioglitazon; *Dendrobium loddigesii* (DLS) groups: IR-like 3T3-L1 cells treated with DLS at 2.03, 4.06, 8.12, and 16.25 μg/mL, respectively; data are means ± SD; ** *p* < 0.01 and *** *p* < 0.001 vs. Mod.

The 3T3-L1 adipocyte was induced in an insulin-resistant (IR) model by dexamethasone and showed the reduction of glucose uptake [31], which is a simple model for screening drugs and is associated with glucose transporter (GLUT) systems [32]. The 2-[*N*-(7-nitrobenz-2-oxa-1,3-diazol-4-yl)amino]-2deoxyglucose (2-NBDG) was used instead of glucose because of its fluorescence. The IR model was used to evaluate the ability of DLS to improve insulin resistance. The effects of DLS on increasing 2-NBDG uptake in 3T3-L1 cells were measured by laser confocal scanning microscopy and the results are shown in Figure 4 and Figure 5.

Figure 4b shows that 2-NBDG uptake in the Mod group was much lower than that in Con group (Figure 4a), which confirmed that the IR model of 3T3-L1 cells was successfully constructed. The strong fluorescence intensity in Figure 4d–g indicates that DLS had the activities of enhancing glucose uptake and reducing insulin resistance in IR-like 3T3-L1 cells. Figure 5 reveals that DLS increased 2-NBDG uptake in a dose-dependent manner. Compared with the Mod group, 2-NBDG uptake was increased by 50% to 100% in 2.03 μg/mL to 16.25 μg/mL groups (*p* < 0.01 or *p* < 0.001). The activity of DLS-enhancing 2-NBDG uptake in the 8.12 μg/mL group was similar to that of the 3.92 μg/mL pioglitazone group.

### 2.3. Effect of a Shihunine-Rich Extract of D. loddigesii on Db/db Mice

Db/db mice (BKS.Cg-Dock7m +/+Leprdb/Nju) were spontaneous mutation diabetic mice and were widely used in a T2DM study, for which the symptoms of glucolipid metabolism disorder were obvious, such as obesity and hyperglycemia [29]. In this study, db/db mice were used for evaluating the antidiabetic effects of DLS. The model group (DM group) was the no-treatment db/db mice, the positive drug group (DMMET130 group) was the db/db mice treated with metformin (MET) at a dose of 130 mg/kg, and the normal control group (control group) was the no-treatment C57BL/6 mice. Three DLS groups were set up, at three dosages of 25, 50, and 100 mg/kg, respectively, which were named DMDLS25, DMDLS50, and DMDLS100 groups, respectively. The results of the animal experiment were as below. 

#### 2.3.1. Effects of a Shihunine-Rich Extract of *D. loddigesii* on Body Weight, Blood Glucose Level, and Oral Glucose Tolerance Test

**Figure 6:**
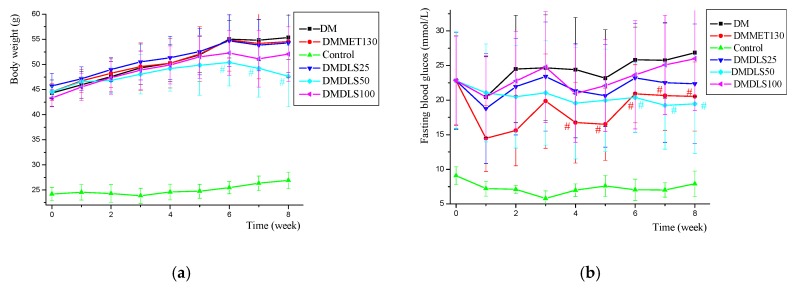
Effects of a shihunine-rich extract of *Dendrobium loddigesii* (DLS) on body weight and fasting blood glucose level across time in the mice. (**a**) Body weight changes over time; (**b**) fasting blood glucose level changes over time. Data are means ± SD; # *p* < 0.05 vs. no-treatment diabetes mellitus (DM) group.

The body weight and fasting blood glucose level of the mice were measured weekly throughout the 8 weeks of DLS treatment. Figure 6a,b present the data tested. 

Figure 6a shows that the body weight of all experimental mice increased gradually, while that of the db/db mice was relatively heavy and that of the control group was comparatively light. The body weights of the DM, DMDLS25, and DMMET130 groups were similar across time, and that of the DMDLS100 group was slightly decreased. Compared with the DM group, the body weight of the DMDLS50 group was significantly reduced after 6 weeks of DLS treatment (*p* < 0.05) and was decreased by 16.4% at the 8th week (*p* < 0.05).

Figure 6b reveals that the fasting blood glucose (FBG) of the control group remained at a low level, and that all db/db mice exhibited a high level that increased gradually. The FBG level of the DM group was the highest and that of the DMDLS25 and DMDLS100 groups was slightly improved after 8 weeks of DLS treatment. Compared to the DM group, the FBG level of the DMMET130 group was significantly reduced from the 4th week. After DLS treatment, the FBG level of the DMDLS50 group improved gradually across time and was significantly reduced from the 6th week (*p* < 0.05 vs. DM group). The FBG level of the DMDLS50 group was reduced by 27.6% compared to the DM group at the 8th week.

**Figure 7:**
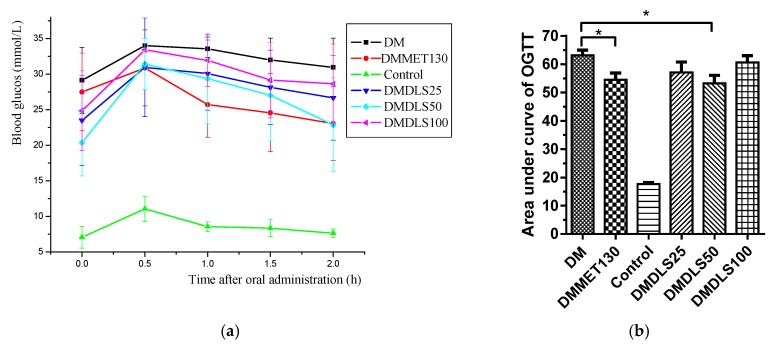
Effect of a shihunine-rich extract of *Dendrobium loddigesii* (DLS) on an oral glucose tolerance test. (**a**) Blood glucose level across time after oral administration in the mice; (**b**) oral glucose tolerance test; data are means ± SD; * *p* < 0.05 vs. no-treatment diabetes mellitus (DM) group.

The activity of DLS in terms of its ability to improve insulin resistance was evaluated by an oral glucose tolerance test (OGTT) at the end of the 7th week. The results are shown in Figure 7.

Figure 7 shows that the OGTT of the control group was the minimum value, and that of the DM group was the maximum value. The OGTT of the DMMET130 group was significantly ameliorated (*p* < 0.05) compared with the DM group, but the OGTT amelioration of the DMDLS25 and DMDLS100 groups was not obvious. Compared to the DM group, the OGTT of the DMDLS50 group was lowered by 18.5% (*p* < 0.05) and showed effective improvement after 8 weeks of treatment, which was close to that of the DMMET130 group. 

#### 2.3.2. Effect of a Shihunine-Rich Extract of *D. loddigesii* on Serum Insulin and Lipid Levels

To observe the effect of DLS-regulating lipid metabolism, the serum levels of TG, total cholesterol (TC), high-density lipoprotein cholesterol (HDL-C), and low-density lipoprotein cholesterol (LDL-C) were determined by ELISA at the end of the 8th week and the results are shown in Table 1. The serum insulin (INS) levels in the mice were measured simultaneously, and the data relating to these levels are also given in Table 1.

As shown in Table 1, the TG level of the control group was the minimum value, and that of the DM group was the maximum value. The TG level of the DMMET130 group exhibited some improvement. The TG levels of all DLS groups were significantly reduced (*p* < 0.05 or *p* < 0.01). DLS at a dose of 50 mg/kg worked the best, and the TG level of the DMDLS50 group was decreased by 43.7% compared to the DM group (*p* < 0.01). 

Table 1 shows that the trends of TC level variation in the serum were the same as those found in the TG levels. The TC level of the DMDLS50 group was significantly reduced by 30.1% compared to the DM group (*p* < 0.01). The HDL-C and LDL-C levels in the mice failed to respond to the treatment of DLS or metformin. 

From Table 1, we can see that the serum INS level in the DM group was relatively low, and that in the control group was relatively high. Compared with the DM group, the serum INS levels in the mice treated with DLS or metformin were significantly increased (*p* < 0.05 or *p* < 0.01). The serum INS level of the DMDLS50 group was increased by 69.2% compared to the DM group (*p* < 0.01). 

#### 2.3.3. Effect of a Shihunine-Rich Extract of *D. loddigesii* on Tissue Forms of Adipose/Pancreas

**Figure 8:**
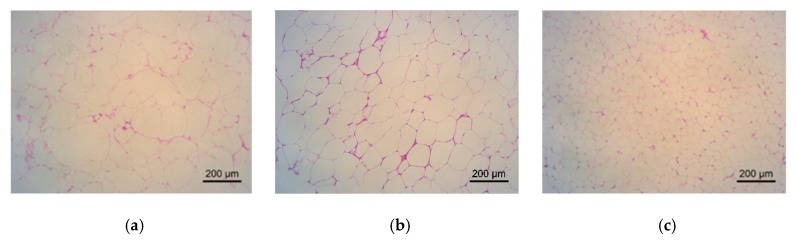

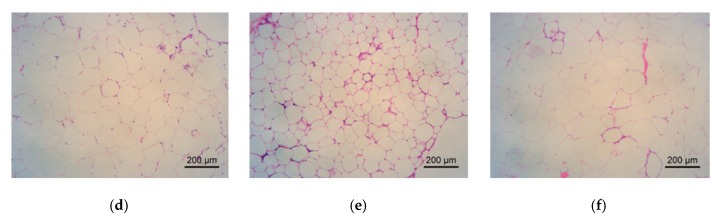
Effect of a shihunine-rich extract of *Dendrobium loddigesii* on morphological changes of adipose tissue in the mice observed under an inverted microscope. (**a**) no-treatment diabetes mellitus (DM) group; (**b**) DMMET130 group; (**c**) no-treatment C57BL/6 mice (control group); (**d**) DMDLS25 group; (**e**) DMDLS50 group; (**f**) DMDLS100 group.

**Figure 9:**
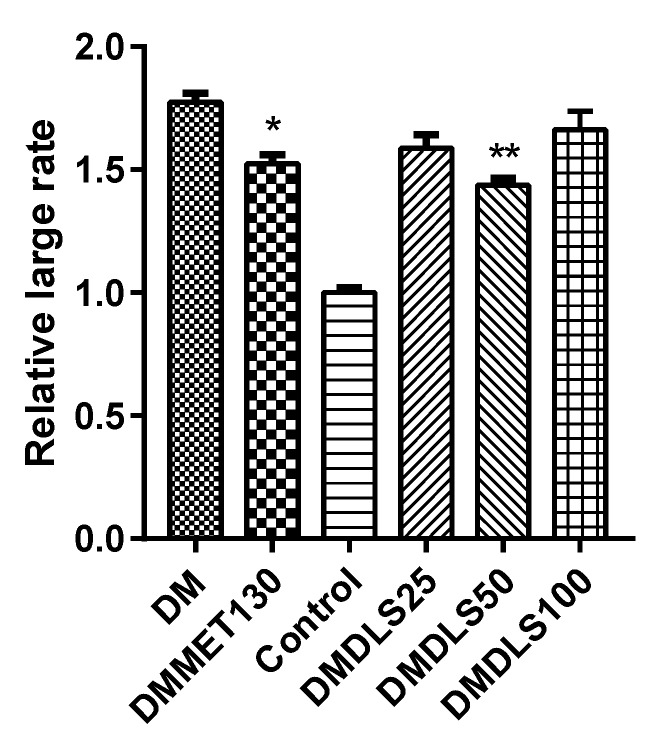
Relatively large rate of adipose cell size in the mice vs. control group. Data are means ± SD; * *p* < 0.05, ** *p* < 0.01 vs. no-treatment diabetes mellitus (DM) group.

The effects of DLS on the adipose tissue of mice were analyzed using hematoxylin-eosin (HE) staining. The results are shown in Figure 8 and Figure 9. 

The adipocyte size in the control group was in a normal state (Figure 8c and Figure 9). In comparison with the control group, the adipocyte size of the DM group increased by 74%, in the DMMET130 group by 53%, in the DMDLS25 group by 59%, in the DMDLS50 group by 44%, and in the DMDLS100 group by 66%. Figure 8 and Figure 9 reveal that the increase in the size of adipocytes in the DMMET130 and DMDLS50 groups was significantly repressed after 8 weeks of treatment with metformin or DLS (*p* < 0.05 or 0.01 vs. DM group). The DMDLS50 group showed the best effect on minifying adipocyte size among the treatment groups.

**Figure 10:**
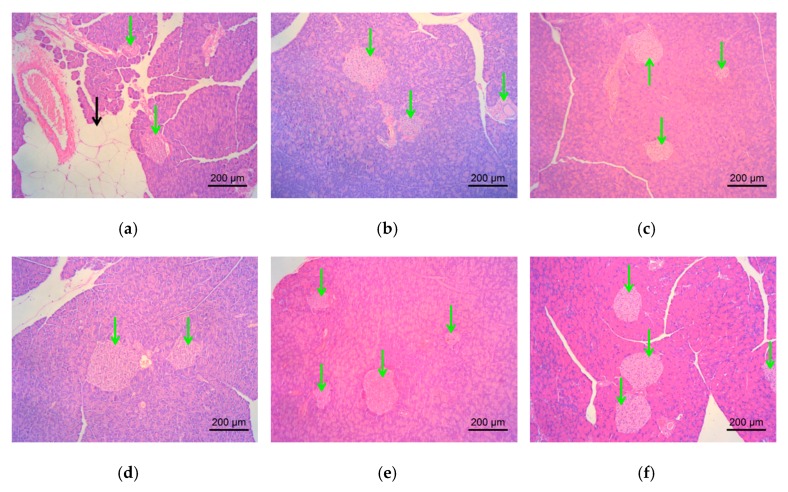
Effect of a shihunine-rich extract of *Dendrobium loddigesii* on the pancreas of mice; the representative areas of adipocyte infiltration are indicated via black arrows; some islets are indicated via green arrows; (**a**) no-treatment diabetes mellitus (DM) group; (**b**) DMMET130 group; (**c**) no-treatment C57BL/6 mice (control group); (**d**) DMDLS25 group; (**e**) DMDLS50 group; (**f**) DMDLS100 group.

**Figure 11:**
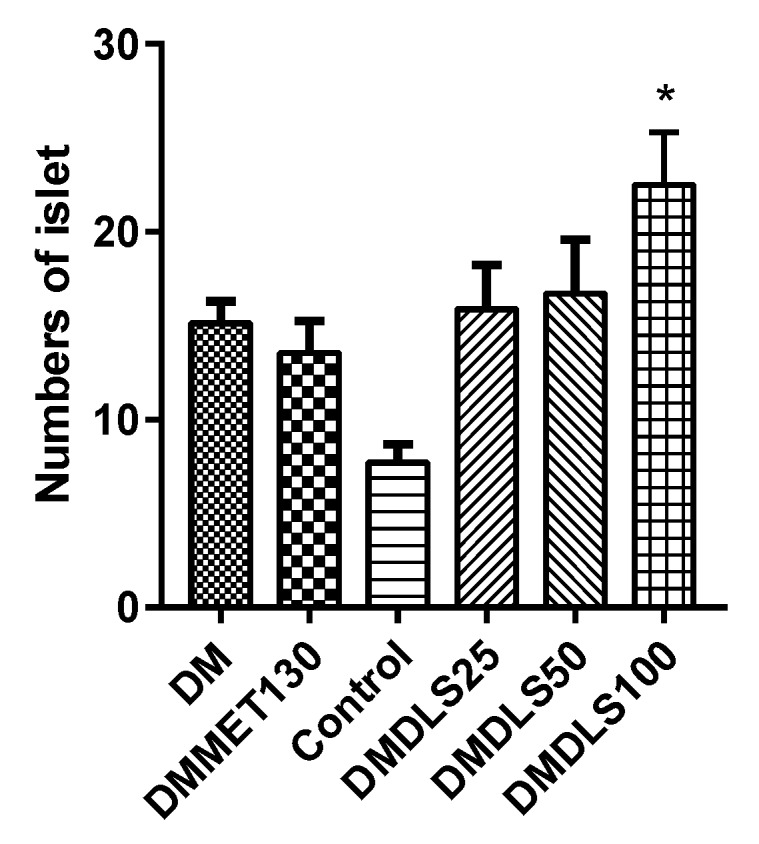
Numbers of pancreatic islets in the mice. Data are means ± SD; * *p* < 0.05 vs. no-treatment diabetes mellitus (DM) group.

The effects of DLS on the pancreatic tissue of the mice were analyzed using HE staining. The paraffin-embedded sections of pancreas are shown in Figure 10, and the numbers of pancreatic islets were counted using Figure 11.

Under an inverted microscope, partial adipocyte infiltration or vacuolar degeneration of the pancreatic cells was observed in the DM group (Figure 10a), which were improved in the mice treated with DLS or metformin. Figure 11 shows that the number of islets in the control group was 8.5, fewer than that of db/db mice (13.57 to 22.5), which might be because C57BL/6 mice and db/db mice (BKS.Cg-Dock7m +/+Leprdb/Nju) are different strains. After 8 weeks of DLS treatment, the islets numbers of DLS groups were increased with a dose–effect relationship, in which the DMDLS100 group displayed the highest numbers of pancreatic islets, 22.5, and significantly higher than those of the DM group (*p* < 0.05).

### 2.4. Effect of a Shihunine-Rich Extract of D. loddigesii on Cleaved Caspase-3 Protein Expression in the Pancreas

**Figure 12:**
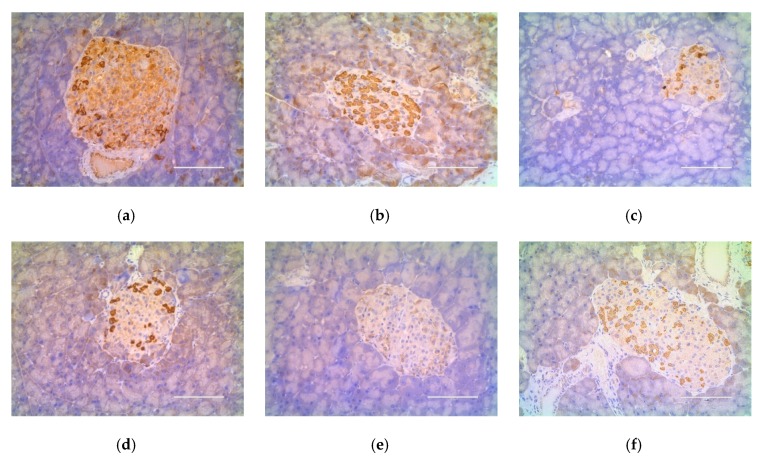
Effect of a shihunine-rich extract of *Dendrobium loddigesii* on cleaved caspase-3 protein expression in the pancreas. Scale bars equal to 100 μm; (**a**) no-treatment diabetes mellitus (DM) group; (**b**) DMMET130 group; (**c**) no-treatment C57BL/6 mice (control group); (**d**) DMDLS25 group; (**e**) DMDLS50 group; (**f**) DMDLS100 group.

**Figure 13:**
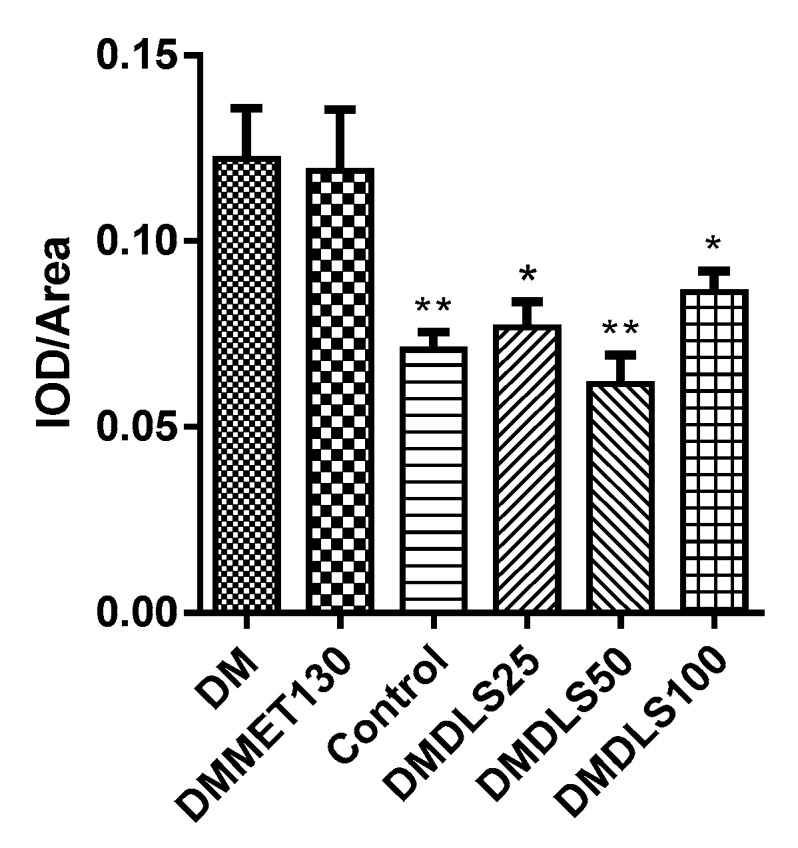
Gray value (IOD/Area) of immunohistochemistry sections of the cleaved caspase-3. Data are means ± SD (*n* = 8–10); * *p* < 0.05 and ** *p* < 0.01 vs. no-treatment diabetes mellitus (DM) group; IOD: integrated optical density.

The expression level of cleaved caspase-3 in the pancreas was measured using immunohistochemical staining to reveal the mechanism by which DLS increased the islet numbers. The sections of immunohistochemistry are shown in Figure 12 and the gray value is shown in Figure 13.

The expression level of cleaved caspase-3 in the DM group was higher than in other groups (Figure 12a and Figure 13). Compared with the DM group, the cleaved caspase-3 expression level in the DMMET130 group was not improved, but that in three DLS groups was dramatically decreased (*p* < 0.05 or 0.01). The cleaved caspase-3 expression level of the DMDLS50 group was similar to that of the control group and decreased by 50% compared to the DM group. 

### 2.5. Effect of a Shihunine-Rich Extract of D. loddigesii on p-AMPK, GLUT4, and PPARα Protein Expressions in Tissue of the Adipose/Liver

**Figure 14:**
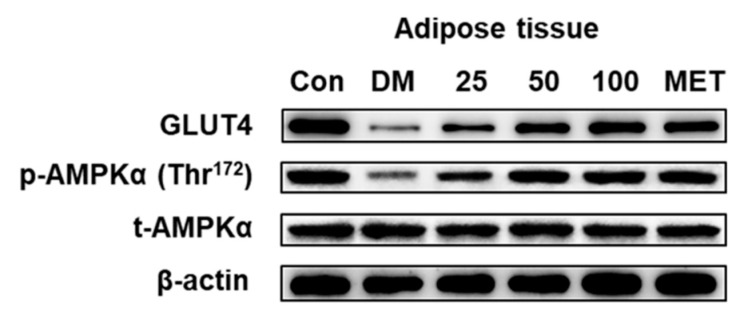
Effects of a shihunine-rich extract of *Dendrobium loddigesii* (DLS) on the protein expressions of GLUT4, p-AMPKα (Thr^172^), and total-AMPKα in adipose tissue by Western blot analysis. β-actin: reference protein; Con: control group, no-treatment C57BL/6 mice; DM: no-treatment db/db mice; MET: DMMET130 group, db/db mice treated with metformin at a dose of 130 mg/kg; 25, 50, and 100: DMDLS25, DMDLS50 and DMDLS100 groups, db/db mice treated with DLS at three dosages of 25, 50, and 100 mg/kg, respectively.

**Figure 15:**
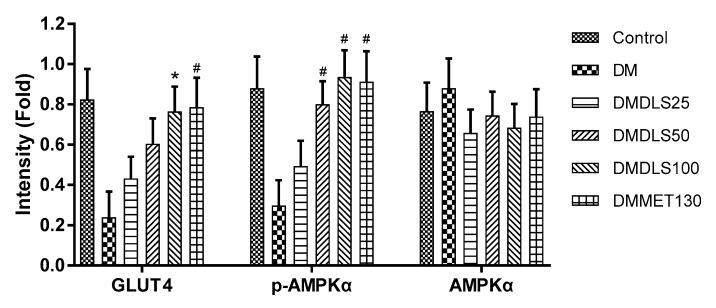
Intensity values of the protein expressions of GLUT4, p-AMPKα, and AMPKα in the adipose tissue. Data are means ± SD; * *p* < 0.01; # *p* < 0.001 vs. no-treatment diabetes mellitus (DM) group.

DLS showed the anti-diabetic activities both at cellular and animal levels. To illuminate the protein factor of the DLS-regulating glucose metabolism, the expressions of p-AMPKα (Thr^172^) and GLUT4 in the adipose tissue were determined by Western blot analysis. The results are shown in Figure 14 and Figure 15. 

Figure 14 and Figure 15 showed that there were no notable differences in total AMPKα expression levels among the six mice groups. Compared with the DM group, the p-AMPKα expressions of the mice were significantly enhanced after 8 weeks of treatment with DLS or metformin (*p* < 0.001). In DLS groups, p-AMPKα protein expressions were dose-dependently enhanced. The p-AMPKα expression levels of DMDLS100 and DMMET130 groups tripled compared to that of the DM group (*p* < 0.001). 

The variation trends of GLUT4 expression in the adipose tissue were similar to those of the p-AMPKα protein. The GLUT4 expression levels in DLS groups were dose-dependently enhanced. The GLUT4 expression levels of the DMDLS100 or DMMET130 groups tripled compared to that of the DM group (*p* < 0.001).

**Figure 16:**
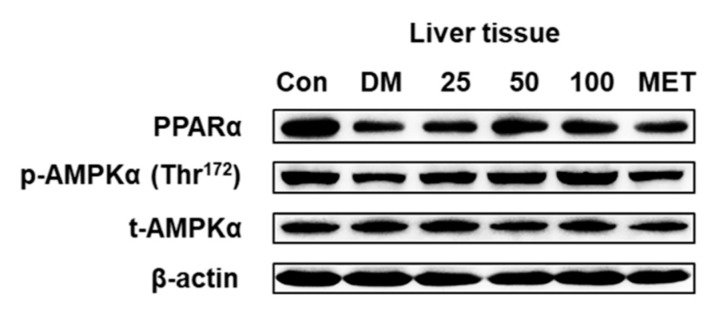
Effects of a shihunine-rich extract of *Dendrobium loddigesii* on the protein expressions of PPARα, p-AMPKα, and AMPKα in the mice liver tissue by Western blot analysis. β-actin: reference protein; Con: control group, no-treatment C57BL/6 mice; DM: no-treatment db/db mice; MET: DMMET130 group, db/db mice treated with metformin at a dose of 130 mg/kg; 25, 50, and 100: DMDLS25, DMDLS50 and DMDLS100 groups, db/db mice treated with DLS at three dosages of 25, 50, and 100 mg/kg, respectively.

**Figure 17:**
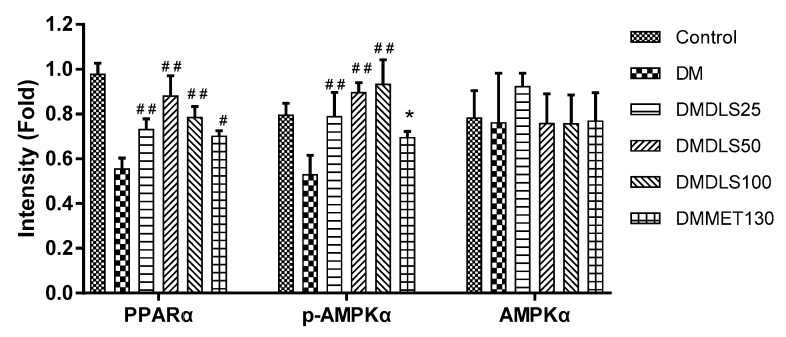
Intensity values of protein expressions of PPARα, p-AMPKα, and AMPKα in the liver tissue. Data are means ± SD; * *p* < 0.05; # *p* < 0.01; ## *p* < 0.001 vs. no-treatment diabetes mellitus (DM) group.

To probe the protein factor of the DLS-regulating lipid metabolism, PPARα and p-AMPK expressions in mice liver tissue were detected. Figure 16 and Figure 17 were the results of Western blot analysis.

Figure 16 and Figure 17 show that the expression levels of total AMPKα protein in the liver tissue were not markedly changed. After 8 weeks of treatment with DLS or metformin, the p-AMPKα expression levels of all drug groups were significantly increased more than that of the DM group (*p* < 0.001 or 0.05). In the DLS groups, the increase in p-AMPKα expression levels showed a dose–effect relationship. Figure 16 and Figure 17 show that the highest expression of PPARα was in the control group, and the PPARα expression levels in the drug groups were significantly increased (p < 0.01 vs. DM group) after 8 weeks of treatment. Additionally, the PPARα expression level of the DMDLS50 group was increased by 34% compared to the DM group.

### 2.6. Toxic Effect of a Shihunine-Rich Extract of D. loddigesii on C57BL/6 Mice

To test the toxicity of a shihunine-rich extract of *D. loddigesii* (DLS), a large dose of DLS, at a dose of 200 mg/kg, was given to C57BL/6 mice (DLS200 group). The serum levels of alanine aminotransferase (ALT), aspartate aminotransferase (AST), uric acid (UA), and creatinine (CRE) in the mice were estimated by ELISA. The results are shown in Table 2.

Table 2 shows that there was no statistical difference in the serum levels of ALT, AST, and CRE between the control C57 group and DLS200 group, but the serum UA level of the DLS200 group was 305.51 μmol/L, which was obviously higher than the 230.23 μmol/L value of the control group (*p* < 0.05). 

A comparison of the sections of gastric mucosa between the control group and DLS200 group revealed that DLS at a dose of 200 mg/kg caused gastric mucosa injury and the numbers of parietal and chief cells were reduced by 21 to 40% in C57 mice (Figure S5). A histology analysis of the gastric mucosa in the db/db mice revealed that DLS at a dose of less than 100 mg/kg improved the gastric mucosa injury and reduction in numbers of parietal and chief cells in the db/db mice (Figure S6). Figure S6a shows that gastric mucosa partial atrophy occurred in the DM group, and their numbers of parietal and chief cells were reduced by 41 to 75%. After 8 weeks of DLS treatment, the gastric mucosa injury was improved, and the numbers of parietal and chief cells were reduced by 21 to 40% in DLS groups. However, the injury of gastric mucosa showed no sign of improvement after metformin treatment at a dose of 130 mg/kg (Figure S6b).

## 3. Discussion

Diabetes mellitus has become a major threat to the health of human beings. Investigations on new antidiabetic agents of nature have attracted the attention of researchers and many natural products with hypoglycemic and antihyperlipidemic properties, such as fenugreek seed, have been extensively studied and used in practice [33]. This research demonstrated that a shihunine-rich extract of *D. loddigesii* (DLS) reduced the accumulation of oil droplets and TG and increased 2-NBDG uptake in 3T3-L1 cells. Further db/db mice experiments verified that DLS reduced the body weight, blood glucose, and serum TG and TC levels and improved the glucose tolerance of the middle dose group (50 mg/kg). The antidiabetic effects of DLS were obviously confirmed in db/db mice and 3T3-L1 cell culture systems, which were close to those of positive drug groups (metformin or pioglitazone). In China, every prescription with herbs is often cooked with water (extracted with water) and DLS is a polar extract of *D. loddigesii* that has excellent water solubility. These findings were useful for understanding the anti-diabetic mechanism of *Dendrobium*. It was the first report on the antidiabetic activity of DLS to our knowledge.

This research revealed that DLS at a dose of less than 50 mg/kg improved the T2DM symptoms of db/db mice and showed a dose–effect relationship (Figure 6, Figure 7, Figure 8 and Figure 9 and Table 1). However, DLS at a dose of 100 mg/kg was relatively ineffective. T2DM is a complex metabolic disorder and many factors influence its development. The biological networks of the experimental mice regulated their homeostasis and symptoms, such as their body weight, blood glucose level, oral glucose tolerance test, and serum insulin and lipid levels, which were the results of the complex regulatory system in the bodies of mice. The complex function of DLS could not be determined. Further studies revealed that DLS at a large dose of 200 mg/kg could cause the obvious increase in serum UA level and gastric mucosa injury in the C57 mice, which hinted that DLS at a dose of 100 mg/kg may have harmful effects on its anti-diabetic properties [34,35].

The anti-apoptotic property of islet cells is thought to be one of the important strategies to treat diabetes mellitus. Cleaved caspase-3 is a critical protein in the process of islet β-cell apoptosis. Pancreatic β-cell apoptosis inhibited has been related to the down regulation of cleaved caspase-3 [36]. Tan reported that geniposide showed antidiabetic effects, and its functionary mechanism was considered to inhibit caspase-3 and caspase-9 protease activity and reduce islet cell apoptosis [37]. This research showed that DLS can increase the serum insulin levels and numbers of islet cells in db/db mice. In DLS groups, it was demonstrated that the expression of cleaved caspase-3 protein in the pancreas was reduced using immunohistochemical analysis. This result suggested that DLS may have the effect of ameliorating the function of islets.

In recent years, the AMPK–GLUT4 pathway and PPARα proteins, as therapeutic targets of T2DM, have drawn great attention from scholars. AMPK activators are natural products and play a role in ameliorating diabetic symptoms through the AMPK–GLUT4 pathway [38]. Resveratrol is a natural phytoalexin that exists widely in many plants and can increase insulin sensitivity and improve insulin resistance. Penumathsa et al. reported that the treatment of resveratrol increased AMPK phosphorylation and regulated Glut-4 translocation, and then increased glucose uptake in diabetic rats [39]. It was reported that berberine reduced lipid accumulation in the 3T3-L1 adipocyte. Additionally, the treatment of berberine lowered the fasting blood glucose level and improved insulin resistance in db/db mice and the related mechanism confirmed that berberine activated the AMPK–GLUT4 signaling pathway [40]. Pu et al. reported that naringin attenuated the obesity, dyslipidemia, fatty liver, liver dysfunction, and insulin resistance on high-fat diet (HFD)-fed mice, and its protection effect worked by phosphorylating AMPKα and insulin receptor substrate 1 (IRS 1) [41]. Ko et al. reported that bergamottin treatment significantly reduced the weight of obese mice by activating AMPK [42]. Ursolic acid is a PPARα activator [43]. Jia et al. reported that ursolic acid reduced the levels of blood glucose and lipid in HFD-fed mice, and the related mechanism was demonstrated that ursolic acid activated the PPARα and induced the hepatic autophagy pathway of the mice [44]. Bak et al. reported that wogonin ameliorated glucose and lipid metabolism by enhancing PPARα expression in the mice [45]. In this research, the results of the anti-diabetic mechanism displayed that DLS enhanced the expression levels of GLUT-4 and p-AMPK in the adipose tissue and increased the expression levels of PPARα and p-AMPK in the liver tissue. Our results suggest that the mechanism of DLS anti-diabetic and relieving dyslipidemia may up-regulate the expressions of p-MAPK, GLUT4, and PPARα. 

## 4. Materials and Methods

### 4.1. Reagents

The ELISA kits of biochemical analyses and Oil Red O dye were purchased from Nanjing Jiancheng Institute of Bioengineering (Nanjing, Jiangsu, China). The bicinchoninic acid (BCA) protein quantitative kit was purchased from Beyotime Biotech Inc. (Shanghai, China). The primary antibodies: cleaved caspase-3 was purchased from Wuhan Servicebio Technology Co., Ltd. (Wuhan, China); AMPKα, p-AMPKα (Thr^172^), and β-actin were purchased from Cell Signaling Technology Inc. (Danvers, MA, USA), and GLUT4 and PPARα were purchased from Santa Cruz Biotechnology, Inc. (Dallas, TX, USA). The secondary antibodies: horseradish peroxidase (HRP) conjugated anti-mouse IgG were purchased from Wuhan Servicebio Technology Co., Ltd. (Wuhan, China); HRP-conjugated affinity purified goat anti-rabbit IgG was purchased from Proteintech Group, Inc. (Rosemont, IL, USA), and m-IgGκ BP-HRP was purchased from Santa Cruz Biotechnology, Inc. (Dallas, TX, USA). The 2-NBDG was purchased from Nanjing Keygen Biotech Co., Ltd. (Nanjing, China), and metformin hydrochloride, pioglitazone hydrochloride, insulin, 3-isobutyl-1-methylxanthine, dexamethasone, and sodium carboxymethylcellulose (CMC–Na) were purchased from Sigma Chemical Co. (St. Louis, MO, USA). Chemical reagents were purchased from Guangzhou Chemical Reagent Factory (Guangzhou, China) and methanol-*d*_4_ (99.9%) was purchased from Aldrich (Milwaukee, WI, USA). Analytical-grade salicylic acid was purchased from Nanjing Reagent (Nanjing, China) and purified by recrystallization with ethanol-H_2_O.

### 4.2. Materials

The 3T3-L1 preadipocytes were purchased from the American Type Culture Collection (Manassas, VA, USA).

The *D. loddigesii* used in this study was the same herbal batch that has been described in the literature [29].

BKS.Cg-Dock7m +/+Leprdb/Nju mice (db/db mice, male, 6–8 weeks old) were purchased from Nanjing Biomedical Research Institute of Nanjing University (Nanjing, China, Approval No. SCXK (SU) 2015-0001) and C57BL/6J mice (male, 6–8 weeks old) were purchased from Laboratory Animal Center of Sun Yat-sen University (Guangzhou, China, Approval No. SCXK (YUE) 2016-0029).

### 4.3. Preparation and Identification of the Shihunine-Rich Extract of D. Loddigesii

The dry stems of *D. loddigesii* (1 kg) were ground and extracted with an acetone–water solution (80:20, *v*/*v*; 3 × 5 L) at room temperature to generate 70 g of crude extract [29]. The crude extract of *D. loddigesii* was loaded on a column of RP-18 gel and washed by water. The water eluate was merged and evaporated to dryness to give a polar extract (31.7 g), forming 3.1% of the weight of raw materials. A shihunine-rich extract of *D. loddigesii* (DLS) was obtained after purifying by a Sephadex LH-20 column (MeOH), repeatedly, and the shihunine structure in DLS was elucidated using a Bruker Avance 400 spectrometer at 400 MHz (Bruker Biospin, Rheinstetten, Germany). A Bruker Avance 500 NMR spectrometer at 500 MHz (Bruker Biospin, Rheinstetten, Germany) was used for qNMR analyses of shihunine in DLS. The qNMR operation has been described in detail in the literature [46] and the method of preparing analysis samples has been described in detail in the literature [29], but the difference was that 10 mg of DLS and 6.0 mg of salicylic acid were weighed out precisely and re-dissolved in 1 mL of methanol-*d*_4_.

### 4.4. Cell Experiments

#### 4.4.1. T3-l1 Cell Culture, Differentiation Induction, and Insulin-Resistant Adipocyte Model

The 3T3-L1 preadipocytes were cultured in complete medium (CM) at 37 °C and 5% CO_2_. CM: Dulbecco’s modified eagle medium containing 10% fetal bovine serum. The differentiation of 3T3-L1 preadipocytes was induced with 10 µg/mL insulin and 0.5 mM 3-isobutyl-1-methylxanthine in CM. The whole stage of induction took more than a week, and detailed operations of each step have been described in the literature [30]. The fully differentiated 3T3-L1 adipocytes were treated with 1 µM dexamethasone for 48 h, and an insulin-resistant (IR) adipocyte model was then established, and detailed operations have been described in the literature [42]. 

After the differentiated 3T3-L1 cells and insulin-resistant adipocytes were treated with 1.02, 2.03, 4.06, 8.12, and 16.25 mg/mL DLS for 48 h, respectively, the cell viability was measured by the Tetrazolium Dye (MTT) method, which showed that the cell viability was not affected by DLS in the experimental range of concentration (Figure S7).

#### 4.4.2. Determination of Intracellular Lipid Content, Triglyceride Content, and Glucose Uptake

After the differentiation of 3T3-L1 cells, intracellular lipid contents were determined using 0.2% (*w*/*v*) Oil Red O for 10 min at room temperature [47] and the images were taken under an FL Auto Imaging System+ EVOS^®^ Onstage Incubator (Life Technologies Corporation, Grand Island, NY, USA). The gray values were calculated using Image-Pro Plus 6.0 (http://www.mediacy.com/, Media Cybernetics, Inc., Rockville, MD, USA).

The analytical procedures of intracellular triglyceride (TG) content are referenced in the literature [48]. An adequate amount of differentiation cells was disrupted with lysate and the supernatant was collected by centrifugation. The TG concentration was evaluated using ELISA kits and the concentration of total protein was determined by a BCA protein assay kit simultaneously. The final TG content was normalized to the concentration of total protein.

After insulin-resistant adipocyte cells were stimulated with 100 nmol/L insulin for 15 min, the cells were cultured in glucose-free culture medium containing 100 μM 2-NBDG for 30 min at 37 °C, and then visualized using a confocal laser scanning microscope Olympus fv3000 (Olympus, Shinjuku, Japan) with 488 nm excitation and 540 nm emission wavelengths. The fluorescence intensity values were calculated using Image J Software 1.6 (National Institutes of Health, Bethesda, MD, USA). A much more detailed operating description has been given in the literature [42].

### 4.5. Animal Experiments

The total of 40 db/db mice and 20 C57 mice used in this study were kept and experimented in a barrier system, which were provided by the Laboratory Animal Center of Sun Yat-sen University (Approval No. SYSK (YUE) 2016-0112). All procedures involving animals were reviewed and approved by the Sun Yat-sen University Institutional Animal Care and Use Committee (IACUC) (Approval No. SYSU-IACUC-2018-000290). The mice were maintained in a controlled light condition (12 h light–dark cycle), the intake of diet and water was completely free, and the diet and water were provided by the Laboratory Animal Center of Sun Yat-sen University.

The db/db mice were randomly divided into five groups and each group had eight mice: (1) DM group: no-treatment diabetes db/db mice; (2) DMMET130 group: metformin (MET) treatment diabetes db/db mice, at a dose of 130 mg/kg; (3) DMDLS25 group: DLS-treatment diabetes db/db mice, at a dose of 25 mg/kg; (4) DMDLS50 group: DLS-treatment diabetes db/db mice, at a dose of 50 mg/kg; and (5) DMDLS100 group: DLS-treatment diabetes db/db mice, at a dose of 100 mg/kg. The C57BL/6 mice were randomly divided into two groups, and each group had 10 mice; (1) control group: no-treatment C57 mice; (2) DLS200 group: DLS-treatment C57 mice, at a dose of 200 mg/kg.

DLS and MET were dissolved in 0.6% CMC–Na solution, respectively, and given to the mice by oral administration. The treatment mice were given drugs once a day for 8 weeks. Non-treatment groups, the DM group, and the control group were given the same volume of 0.6% CMC–Na solution by oral administration once a day for 8 weeks. Body weight and fasting blood glucose (FBS) of the mice were measured once a week. An oral glucose tolerance test (OGTT) was performed in the seventh week. At the end of the experiment, blood was taken from the mice for the preparation of serum, and the tissues of the mice were collected and frozen in liquid nitrogen or placed in 10% formalin solution for further analysis. 

### 4.6. Blood Glucose and Serum Biochemical Indices Analysis

The detection of FBS and the OGTT were performed using the glucose meter Accu-Chek Performa (Roche Diagnostics GmbH, Mannheim, Germany), as described previously [29].

The serum biochemical indices of the mice were determined using the ELISA kit based on the manufacturer’s instructions. A multi-mode microplate reader Flex Station 3 (Molecular devices, Sunnyvale, CA, USA) was used for absorbance testing.

### 4.7. Histologic Analysis and Immunohistochemical Analysis

The histologic analysis of the mice was performed as described previously [29]. The change of histomorphology was observed using a LEICA DM5000B microscope (Leica, Heidelberg, Germany).

The immunohistochemical analysis of cleaved caspase-3 in the pancreas was performed as described previously [29]. The images were taken under a FL Auto Imaging System+ EVOS^®^ Onstage Incubator (Life Technologies Corporation, Grand Island, NY, USA). The gray values were calculated using Image-Pro Plus 6.0 (Media Cybernetics, Inc., Rockville, MD, USA).

### 4.8. Western Blot Analysis

Adipose and liver tissue samples were lysed with ice-cold lysis buffer including proteinase inhibitor, and the protein contents were determined using the BCA protein assay kit. After the proteins were quantified, Western blot analysis was performed, as described in the literature [49]. Densitometry values for Western blot analysis were analyzed by Image J Software 1.6 (National Institutes of Health, Bethesda, MD, USA).

## 5. Conclusions

This study revealed that a shihunine-rich extract of *D. loddigesii* (DLS) is an important anti-diabetic and lipid-lowering component of *D. loddigesii*, which might be the result of the synergistic effects of all components in DLS. DLS at a dose of 50 mg/kg has good anti-diabetic efficacy in db/db mice and fewer side effects.

## Figures and Tables

**Table 1:** Effects of a shihunine-rich extract of *Dendrobium loddigesii* on triglyceride (TG), total cholesterol (TC), high-density lipoprotein cholesterol (HDL-C), low-density lipoprotein cholesterol (LDL-C), and insulin (INS) levels in the mice serum.

Group	TG	TC	HDL-C	LDL-C	INS
	mmol/L				mIU/L
DM	3.34 ± 1.25	7.42 ± 1.49	2.27 ± 0.44	0.79 ± 0.29	13.12 ± 2.81
DMMET130	2.49 ± 0.73	7.08 ± 1.10	2.53 ± 0.63	1.14 ± 0.48	21.29 ± 7.68 *
Control	1.06 ± 0.33	3.52 ± 0.64	2.11 ± 0.17	0.22 ± 0.03	25.12 ± 4.81
DMDLS25	2.13 ± 0.40 *	6.11 ± 1.25	2.64 ± 0.48	0.97 ± 0.38	20.06 ± 4.94 *
DMDLS50	1.88 ± 0.31 **	5.19 ± 1.68 **	2.19 ± 0.51	0.83 ± 0.34	22.21 ± 4.39 **
DMDLS100	1.90 ± 0.23 **	6.73 ± 0.71	2.20 ± 0.23	1.00 ± 0.34	17.55 ± 2.62

Data are means ± SD; * *p* < 0.05, ** *p* < 0.01 vs. no-treatment diabetes mellitus (DM) group.

**Table 2:** Effects of shihunine on the serum levels of alanine aminotransferase (ALT), aspartate aminotransferase (AST), uric acid (UA), and creatinine (CRE) in C57 mice.

Group	ALT	AST	UA	CRE
	U/grot		μmol/L	
Control	21.33 ± 11.10	33.27 ± 14.82	230.23 ± 77.76	17.01 ± 7.67
DLS200	28.21 ± 11.86	27.00 ± 11.88	305.51 ± 39.20 *	20.38 ± 5.90

Data are means ± SD; * *p* < 0.05 vs. control group.

## Supplementary Materials

The following are available online: Figure S1: Structure of shihunine, Figure S2: ^1^H-NMR for shihunine from *D. loddigesii*, Figure S3: ^1^H-NMR for qNMR of a shihunine-rich extract of *D. loddigesii* (DLS), Figure S4: ^1^H-NMR of salicylic acid for the external standard, Figure S5: Effect of a shihunine-rich extract of *D. loddigesii* on gastric mucosa morphological changes in C57 mice, Figure S6: Effect of a shihunine-rich extract of *D. loddigesii* on gastric mucosa morphological changes in db/db mice, Figure S7: Effect of a shihunine-rich extract of *D. loddigesii* on the viability of differentiated 3T3-L1 cells.

## Abbreviations

AMPK	adenosine monophosphate-activated protein kinase
ALT	alanine aminotransferase
AST	aspartate aminotransferase
control	no-treatment C57 mice
cleaved caspase-3	cleaved cysteine aspartic acid-specific protease 3
DLS	shihunine-rich extract of *Dendrobium loddigesii*
DLS200	DLS-treatment C57 mice, in a dose of 200 mg/kg
DM	no-treatment diabetes db/db mice
DMDLS100	DLS-treatment diabetes db/db mice, in a dose of 100 mg/kg
DMDLS25	DLS-treatment diabetes db/db mice, in a dose of 25 mg/kg
DMDLS50	DLS-treatment diabetes db/db mice, in a dose of 50 mg/kg
DMMET130	MET-treatment diabetes db/db mice, in a dose of 130 mg/kg
FBG	fasting blood glucose
GLUT4	glucose transporter 4
HDL-C	high-density lipoprotein cholesterol
INS	insulin
IOD	integrated optical density
LDL-C	low-density lipoprotein cholesterol
MET	metformin
OGTT	oral glucose tolerance test
p-AMPK	adenosine monophosphate-activated protein kinase phosphorylation
PPARα	peroxisome proliferator-activated receptor α
T2DM	type 2 diabetes mellitus
TC	total cholesterol
TG	triglyceride
UA	uric acid
CRE	creatinine
