# Supplementary material for: Anti-Diabetic Effect of a Shihunine-Rich Extract of Dendrobium loddigesii on 3T3-L1 Cells and db/db Mice by Up-Regulating AMPK–GLUT4–PPARα
Source: Molecules. 2019 Jul 23;24(14):2673. doi: 10.3390/molecules24142673 (PMC6680686; doi:10.3390/molecules24142673)
Supplement: Supplementary file 1 [file molecules-24-02673-s001.pdf]

Supplementary Materials

Anti-Diabetic Effect of a Shihunine-Rich Extract of *Dendrobium loddigesii* on 3T3-L1 Cells and db/db Mice by Up-Regulating AMPK/GLUT4 /PPARα

Xue-Wen Li [1†], Meixiang Huang [1†], Kakei Lo [1], Wei-Li Chen [1], Ying-Yan He [1], Yongli Xu [1], Huizhen Zheng [1], Haiyan Hu [1,*] and Jun Wang [1,*]

[1] School of Pharmaceutical Sciences, Sun Yat-sen University, Guangzhou, 510006, China
[*] Correspondence: lsshhy@mail.sysu.edu.cn (H.H.); wjun@mail.sysu.edu.cn (J.W.); Tel.: +86-203-933-6119 (H.-Y.H); +86-203-994-3090 (J.W.)

[†] Those authors contribute equally to this work.

Figure Legends:

Figure S1. Structure of shihunine

Figure S2. ^{1}H NMR for shihunine from *D. loddigesii*

Figure S3. ^{1}H NMR for qNMR of shihunine-rich extract of *D. loddigesii* (DLS)

Figure S4. ^{1}H NMR of salicylic acid for the external standard

Figure S5. Effect of shihunine-rich extract of *D. loddigesii* on gastric mucosa morphological changes in C57 mice

Figure S6. Effect of shihunine-rich extract of *D. loddigesii* on gastric mucosa morphological changes in db/db mice

Figure S7. Effect of shihunine-rich extract of *D. loddigesii* on the cell viability of 3T3-L1 preadipocytes

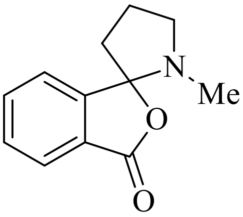

Figure S1. Structure of shihunine

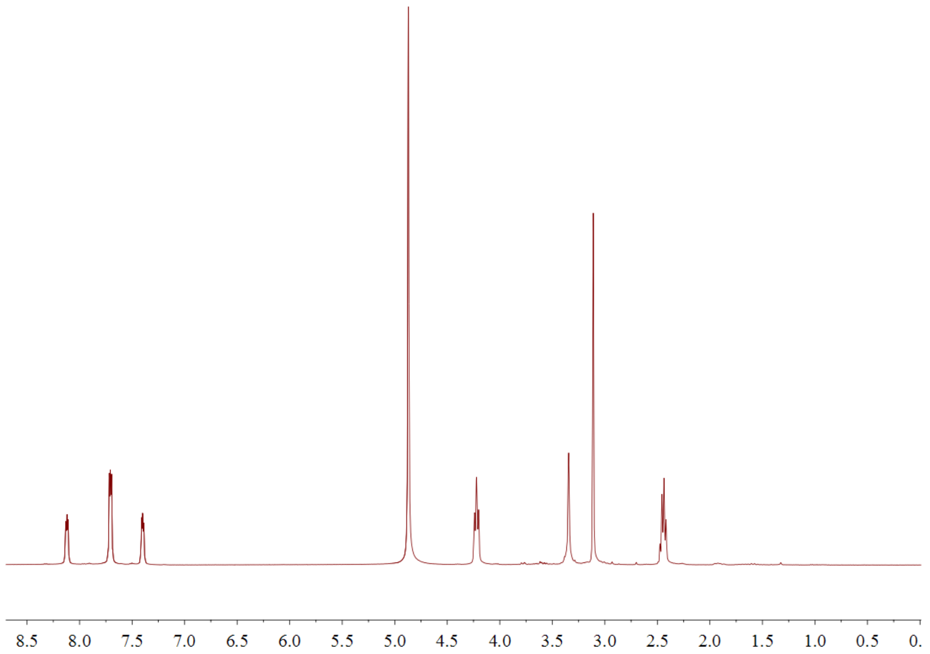

Figure S2. ^{1}H NMR for shihunine from *D. loddigesii*

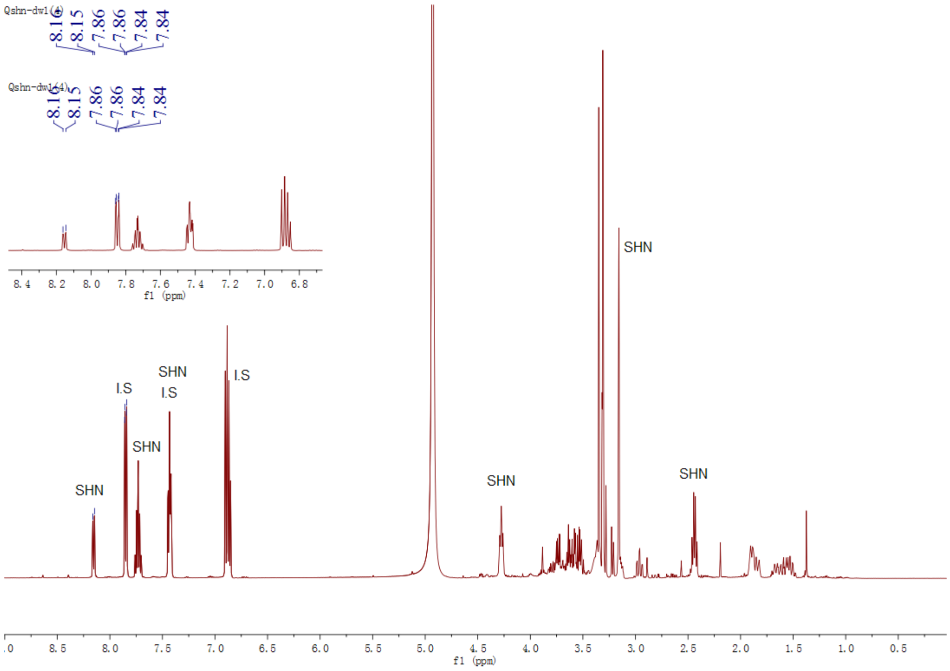

Figure S3. ^{1}H NMR for qNMR of shihunine-rich extract of *D. loddigesii* (DLS); SHN: shihunine; I.S: salicylic acid

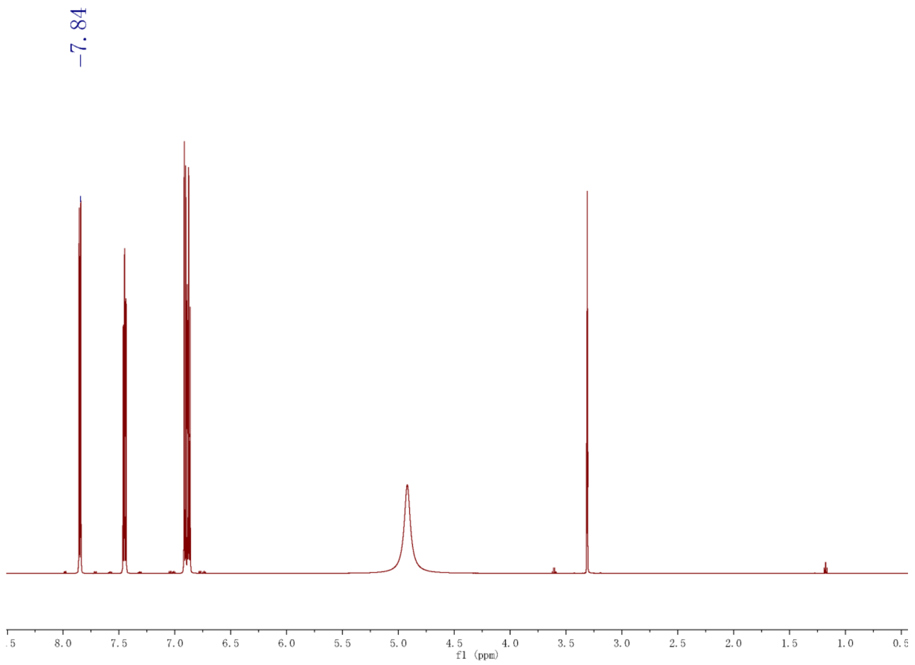

Figure S4. ^{1}H NMR of salicylic acid for an external standard

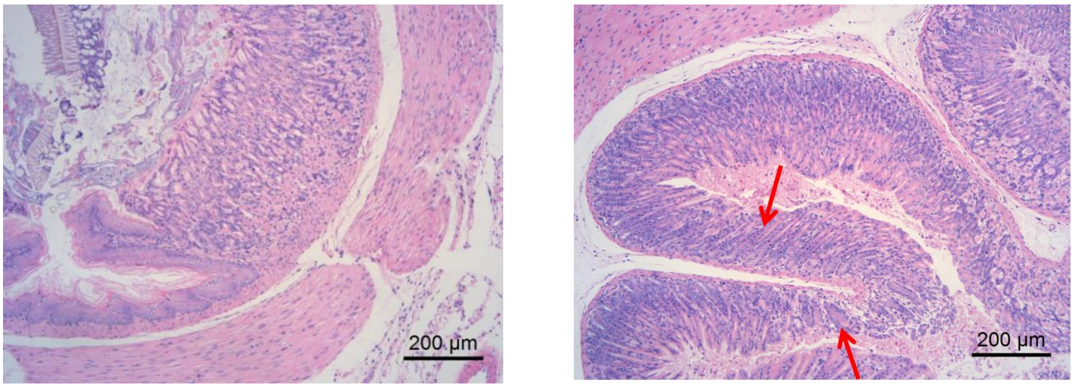

(**a**) (**b**)

Figure S5. Effect of shihunine-rich extract of *D. loddigesii* (DLS) on the gastric mucosa morphological changes in the C57 mice. The atrophy areas of gastric mucosa were indicated via red arrows

(**a**): Control group, no-treatment C57BL/6 mice, which gastric mucosa was in normal states.
(**b**): DLS200 group, DLS-treatment C57BL/6 mice at a dose of 200 mg/kg, which gastric mucosa was partial atrophy; its numbers of main and parietal cells were decreased by 21% to 40%.

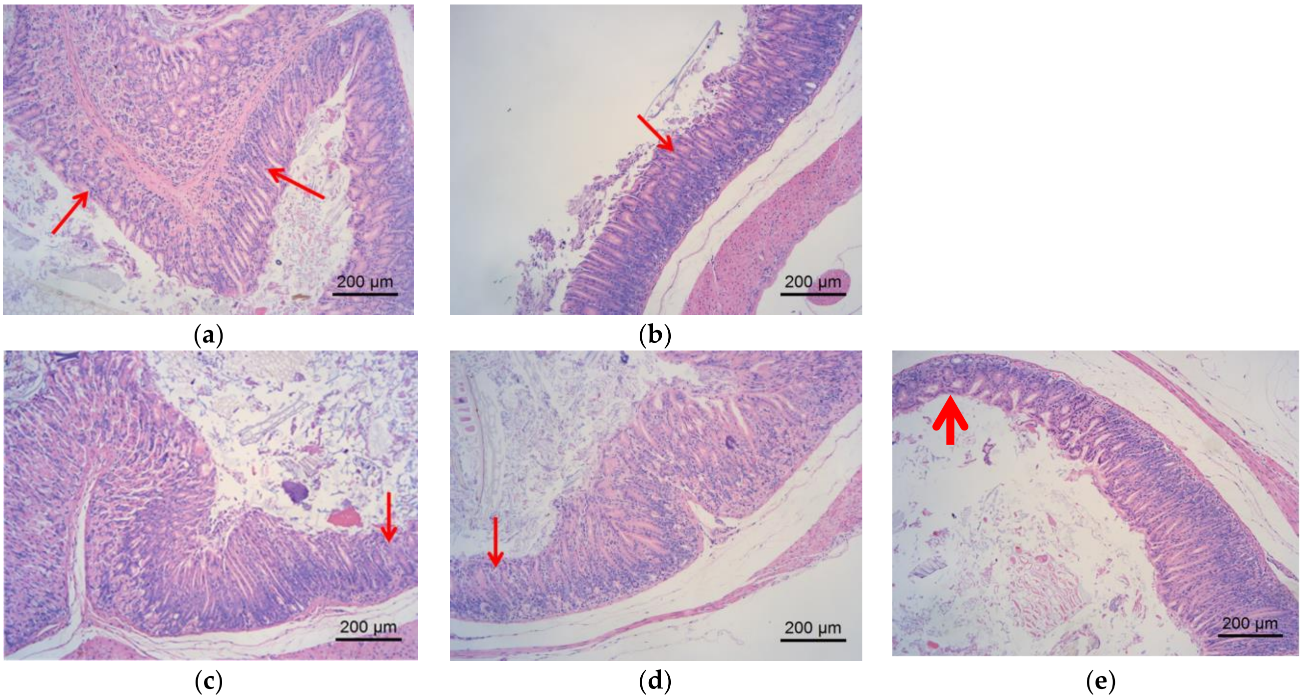

Figure S6. Effect of shihunine-rich extract of *D. loddigesii* (DLS) on the gastric mucosa morphological changes in db/db mice. The atrophy areas of gastric mucosa were indicated via red arrows

(**a**): MD group, no-treatment db/db mice, which gastric mucosa was partial atrophy, and the numbers of main and parietal cell were decreased by 41% to 75%..
(**b**): MDMET130 group, metformin-treatment db/db mice at a dose of 130 mg/kg, which gastric mucosa was partial atrophy, and the numbers of main and parietal cells were decreased by 41% to 75%.
(**c**): MDDLS25 group, DLS-treatment db/db mice at a dose of 25 mg/kg, which gastric mucosa was partial atrophy, and the numbers of main and parietal cells were decreased by 21% to 40%.
(**d**): MDDLS50 group, DLS-treatment db/db mice at a dose of 50 mg/kg, which gastric mucosa was partial atrophy, and the numbers of main and parietal cells were decreased by 21% to 40%.
(**e**): MDDLS100 group, DLS-treatment db/db mice at a dose of 100 mg/kg, which gastric mucosa was partial atrophy, and the numbers of main and parietal cells were decreased by 21% to 40%.

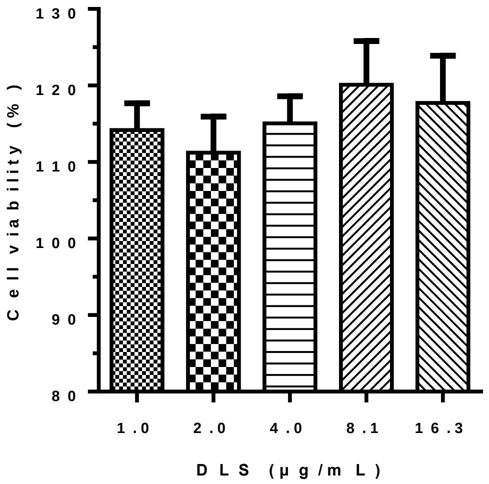

Figure S7. Effect of shihunine-rich extract of *D. loddigesii* (DLS) on the cell viability of 3T3-L1 preadipocytes
